# Intraperitoneal Alpha-Radioimmunotherapy of Advanced Ovarian Cancer in Nude Mice Using Different High Specific Activities

**DOI:** 10.4021/wjon2010.05.208w

**Published:** 2010-05-19

**Authors:** Jorgen Elgqvist, Daniel Ahlberg, Hakan Andersson, Holger Jensen, Bengt R Johansson, Helena Kahu, Marita Olsson, Sture Lindegren

**Affiliations:** a Institute of Clinical Sciences, Sahlgrenska Academy, University of Gothenburg, Sweden; b Mathematical Sciences, University of Gothenburg and Chalmers University of Technology, Sweden; c PET and Cyclotron Unit, Rigshospitalet, Copenhagen, Denmark; d Electron Microscopy Unit, Institute of Biomedicine, Sahlgrenska Academy, University of Gothenburg, Sweden

**Keywords:** Radioimmunotherapy, Alpha, ^211^At, Astatine, Specific activity, Intraperitoneal, Ovarian cancer, Mice

## Abstract

**Background:**

The aim of this study was to investigate the therapeutic efficacy of advanced ovarian cancer in mice, using α-radioimmunotherapy with different high specific activities. The study was performed using the monoclonal antibody (mAb) MX35 F(ab′)2 labeled with the α-particle emitter ^211^At.

**Methods:**

Animals were intraperitoneally inoculated with ≥1 × 10^7^ cells of the ovarian cancer cell line NIH:OVCAR-3. Four weeks later 9 groups of animals were given 25, 50, or 400 kBq ^211^At-MX35 F(ab′)2 with specific activities equal to 1/80, 1/500, or 1/1200 (^211^At atom/number of mAbs) for every activity level respectively (*n* = 10 in each group). As controls, animals were given PBS or unlabeled MX35 F(ab′)2 in PBS (*n* = 10 in each group). Eight weeks after treatment the animals were sacrificed and the presence of macroscopic tumors was determined by meticulous ocular examination of the abdominal cavity. Cumulated activity and absorbed dose calculations on tumor cells and tumors were performed using in house developed program. Specimens for scanning electron-microscopy analysis were collected from the peritoneum at the time of dissection.

**Results:**

Summing over the different activity levels (25, 50, and 400 kBq ^211^At-MX35 F(ab′)2) the number of animals with macroscopic tumors was 13, 17, and 22 (*n* = 30 for each group) for the specific activities equal to 1/80, 1/500, or 1/1200, respectively. Logistic-regression analysis showed a significant trend that higher specific activity means less probability for macroscopic tumors (*P* = 0.02).

**Conclusions:**

Increasing the specific activity indicates a way to enhance the therapeutic outcome of advanced ovarian cancer, regarding macroscopic tumors. Further studies of the role of the specific activity are therefore justified.

## Introduction

Ovarian cancer frequently recurs from remaining micrometastatic growth on the peritoneal surface in spite of debulking surgery and systemic chemotherapy. External abdominal radiotherapy has proven unsuccessful due to absorbed dose limitations in normal tissues. Therefore, adjuvant locoregional treatment with intraperitoneal administration of specific antibodies could be decisive in the treatment of remaining micrometastatic disease. Generally, clinical application of ^211^At-labeled mAbs is a promising research field and has to be investigated further [1]. In this study we used the monoclonal antibody (mAb) MX35 F(ab′)2, which recognizes the sodium dependant phosphate transport protein 2b (NaPi2b) of 90 kDa on ovarian cancer cells. The cytotoxicity was mediated by labeling the mAbs with an α-particle-emitting radionuclide, ^211^At. We used an animal model mimicking the clinical situation with intraperitoneal radioimmunotherapy (RIT). The intraperitoneal approach allows a high absorbed dose to non-vascularized peritoneal tumors with low myelotoxicity as the clearance rate from the peritoneal cavity to the systemic circulation is delayed [2].

Studies have been performed using RIT against ovarian cancer, mostly mAbs labeled with ^90^Y, ^177^Lu, or ^131^I in animals [3-14] and in humans [7, 15-20]. The β-emitting radionuclides have too long a range for the treatment of microscopic tumors [21], thus we believe it is important to continue the investigations of the efficacy of mAbs labeled with α-particle emitters when treating microscopic disease on the peritoneum. In this study, as in a series of earlier studies [22-29], we used the α-particle emitter ^211^At, with a half-life of 7.2 h, a mean range in tissue of 62 µm, and a mean linear energy transfer (LET) of 111 keV/µm. The half-life of this radionuclide makes it ideal for local treatment as the target cells are easily reached while the transfer of the radioimmunocomplex to the systemic circulation is delayed. The short range ensures a significant absorbed dose in microscopic tumors or even single tumor cells. The high LET, together with the high relative biological effectiveness (RBE) of the α-particles necessitating only a few hits to kill the cell, indicates that only a small number of ^211^At-atoms have to be targeted to each cell [2, 30, 31].

As indicated in earlier studies the specific activity could be a decisive parameter deciding whether a treatment will be successful or not [25, 28]. The advantages with administering a radioimmunocomplex with a high specific activity we believe are: (*i*) the ability to treat low antigen expressing tumor and/or tumors having restricted diffusion; and (*ii*) the ability to treat more patients per unit radioactivity. A new radiolabeling technique enables us now to substantially achieve higher specific activities [32]. Therefore, the aim of the study was to investigate the therapeutic efficacy of α-RIT of ovarian cancer in nude mice using different high specific activities. The study is the first one investigating the role of increasing the specific activity during RIT. The study was performed using the mAb MX35 F(ab′)2 labeled with the α-particle emitter ^211^At.

A phase I study on women, with recurrent epithelial ovarian cancer after second-line chemotherapy treated with ^211^At-MX35 F(ab′)2, was published in 2009 [33]. Since we now are planning for a phase II study, intended as a “boost” treatment for women having had cytoreductive surgery and first-line chemotherapy, it is of outmost importance to investigate different aspects of the therapeutic efficacy, among them the role of higher specific activities.

## Materials and Methods

### Radionuclide

^211^At was produced by the ^209^Bi(α, 2n)^211^At reaction in a cyclotron (Scanditronix MC32 at the Positron Emission Tomography and Cyclotron Unit, Rigshospitalet, Copenhagen, Denmark) by irradiating a ^209^Bi target with 28-MeV α-particles. The ^211^At was isolated using a dry-distillation procedure [34].

### Monoclonal Antibodies

MX35 is a murine IgG1-class mAb, developed and characterized at the Memorial Sloan-Kettering Cancer Center (MSKCC), New York, USA. MX35 is directed towards the sodium dependant phosphate transport protein 2b (NaPi2b) of 90 kDa on OVCAR-3 cells [35] and is expressed strongly and homogeneously on 90% of human epithelial ovarian cancers [36]. A batch of MX35 F(ab′)2, produced by Strategic BioSolutions (Newark, USA) for clinical use, was provided by MSKCC.

### Antibody Conjugation and Radiolabeling

The MX35 F(ab′)2 antibody was labeled via the intermediate labeling reagent *N*-succinimidyl-3-(trimethylstannyl)benzoate (m-MeATE) [32]. A stock solution of m-MeATE was prepared by dissolving the 50 mg batch from the supplier Toronto Research Chemicals Inc. (North York, Canada) in 1 mL of chloroform. From the stock solution was taken 2 µL (100 µg, 0.26 µmole) and the chloroform was evaporated. The reagent was redissolved in 20 µL of dimethylsulphoxide (DMSO). To 1 mg of antibody at a concentration of 2.87 mg/mL in 0.2 M carbonate buffer pH 8.5 was added 4 µL of the m-MeATE/DMSO preparation under vigorous agitation. The reaction was allowed to proceed for 30 min at room temperature under gentle agitation. The resulting ε-lysyl-3-(trimethylstannyl)benzamide-MX35 F(ab′)2 immunoconjugate was then isolated from low un-reacted molecular species by size exclusion chromatography on a NAP-5 column (GE-Healthcare, Sweden) using 0.2 M acetate buffer (pH 5.5) as mobile phase.

The produced immunoconjugate was labeled with ^211^At and prepared at three levels of specific radioactivity. To a dry residue of ^211^At (241 MBq) was added 20 µL of N-iodosuccinimide (NIS), 50 µg of MX35 F(ab′)2-lysyl-3-(trimethylstannyl)benzoate at a concentration of 0.25 mg/ml in 0.2 M acetate buffer pH 5.5. After 1 min reaction time 3 µL of NIS (20 µg/mL) in methanol/1% HAc was added to iodosubstitute any remaining stannyl groups on the antibody. After an additional 1 min reaction time the reaction was stopped with an access of sodium metasulfite. The antibody fraction was finely isolated from low molecular weight species by size exclusion chromatography on a NAP-5 column. After labeling nine activity injection solutions were prepared from the labeled stock preparation, 25, 50, and 400 kBq at a specific activity of 1920, 500 or 120 MBq/mg, corresponding to 1/80, 1/500, and 1/1200 ^211^At atoms/mAbs, respectively.

### Cell Line

The cell line OVCAR-3 (NIH:OVCAR-3: National Institutes of Health ovarian cancer cell line 3, USA) was used [37]. The cell line was obtained from the American Type Culture Collection, Rockville, MD, USA. The cells were cultured in T-75 culture flasks at 37°C in a humidified atmosphere of 95% O_2_/5% CO_2_ with RPMI-1640 cell culture medium supplemented with 10% fetal calf serum, 1% L-glutamine and 1% penicillin-streptomycin.

### Immunoreactivity of Antibodies

For determination of the immunoreactive fraction, a single-cell suspension of NIH:OVCAR-3 cells was prepared at a concentration of 5 × 10^6^ cells/mL. The cells were serially diluted, 1 : 2, and a constant amount, 5 ng, of ^211^At-MX35 F(ab′)2 was added to each dilution. The ^211^At-MX35 F(ab′)2 was reacted with the cells for 3 h at room temperature during gentle agitation. After incubation and repeated washing, the bound fraction of each dilution was determined by measuring the activity of the cells. Double inverse plots were derived from the data and the immunoreactive fraction calculated [38]. Nonspecific binding of the astatinated MX35 F(ab′)2 was examined by saturating the antigens on the OVCAR-3 cells with an excess of unlabeled MX35 F(ab′)2.

### Cumulated Activity and Absorbed Dose

**Figure 1:**
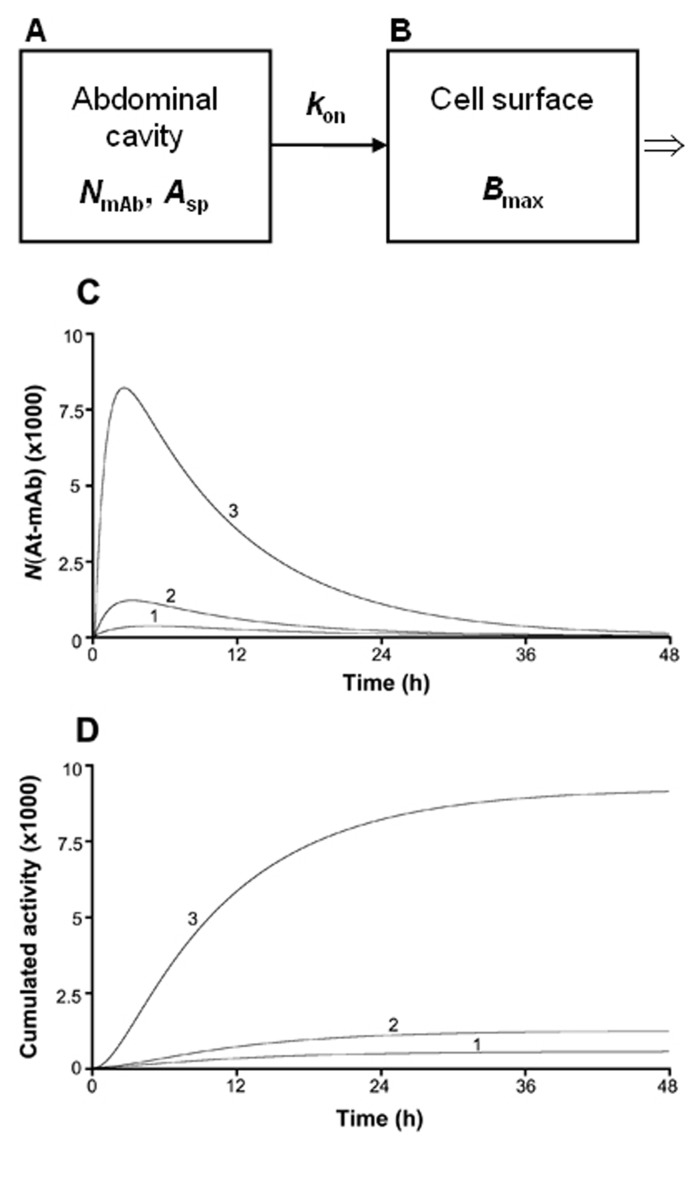
The computational model used for investigating of how the cumulated activity on a tumor cell surface varies for different specific activities (*A*_sp_). *N*_mAb_ in compartment A is the total number of mAbs injected in the abdominal cavity. *k*_on_ is the rate at which the mAbs are bound to the antigenic sites on the cell surface per unit time for a specific antigen concentration, and *B*_max_ in compartment B is the total number of antigens present on one tumor cell. *B*_max_ was in the above calculations set to 10^6^, a value previously determined *in vitro* for the OVCAR-3 cells. *N*_At-mAb_ is the number of ^211^At atoms situated on the tumor cell surface at certain times after injection. Curve 1, 2, and 3 in panel C and D corresponds to a specific activity equal to 1/1200, 1/500, and 1/80 ^211^At atom/mAbs, respectively.

A previously developed dynamic compartment model, which enables the computation of the cumulated activity on a tumor cell, was used [25]. Two compartments were defined: one representing the injected volume and the number of mAbs in the abdominal cavity, and one representing the number of mAbs bound to the antigenic sites of one tumor cell (Fig. 1). The initial value in the first compartment was *N*_mAb_-that is, the total number of intraperitoneally injected mAbs at t = 0. The transition of mAbs from the first compartment to the second compartment was determined using the previously *in vitro* determined *k*_on_-that is, the rate at which mAbs are bound to the antigenic sites on the cell surface per unit time for a specific antigen concentration. The effect of the gradual saturation of the antigenic sites with time was considered by introducing a gradual reduction in the number of free sites from *B*_max_ to zero into the calculations. According to the conditions during the determination of the kinetic and equilibrium constants, the dissociation rate constant, *k*_off_, was negligible and therefore set to zero. During the period of binding and irradiation, a constant concentration of mAbs in the abdominal fluid was assumed. For determination of the cumulated activity on a cell, originating from cell surface-bound mAbs, the number of ^211^At atoms on the cell surface at different times (*N*_At-mAb_) was calculated using the different values of the specific activity, *A*_sp_ (1/80, 1/500, or 1/1200 ^211^At atom/mAbs).

The specific energy imparted to cell nuclei situated at different depths in tumors was calculated using a Monte-Carlo program presented in an earlier paper [26]. The calculations in this study were performed for: 3 tumor sizes; 30, 45, and 95 µm radius, 3 activity levels; 25, 50, and 400 kBq ^211^At MX35 F(ab′)2, 3 specific activity levels; 1/80, 1/500, and 1/1200 ^211^At atom/number of mAbs, and assuming a diffusion depth of 30 µm into the tumors, previously shown to be the activity distribution most consistent with the therapeutic results [27].

### Animals

We used 110 female, nude Balb/c *nu/nu* mice (Charles River Laboratories International Inc., Wilmington, MA, USA) in this study. The animals were housed at 22°C and 50%-60% humidity with a light/dark cycle of 12 h. They were given autoclaved standard pellets and water *ad libitum*. All the experiments were approved by the Ethics Committee of the University of Gothenburg.

### In vivo Procedures and Study Groups

At the age of 5 weeks all mice were intraperitoneally inoculated with ≥1 × 10^7^ OVCAR-3 cells suspended in 0.2 mL saline. Four weeks after cell inoculation the animals were divided into 11 groups. The animals in groups 1-9 were intraperitoneally injected with 25, 50, or 400 kBq ^211^At-MX35 F(ab′)2 in 1 mL saline using specific activities equal to 1/80, 1/500, or 1/1200 (^211^At atom/mAbs) for each activity level, respectively (*n* = 10 in each group). As controls (group 10 and 11), animals were treated with PBS or unlabeled MX35 F(ab′)2 in PBS (*n* = 10 in each group). The animals were weighed weekly. Eight weeks after the intraperitoneal treatment, i.e. 12 wk after cell inoculation, all animals were sacrificed by cervical dislocation and dissected. The abdominal cavity was opened and the presence of ascites, and micro- and macroscopic tumors was determined as “yes” or “no”. Animals dissected and judged were blinded from knowledge from exposure conditions.

### Scanning Electron Microscopy

Specimens for ultrastructural analysis were obtained from mice anesthetized with Metofane (Mallinckrodt Veterinay Inc.) at the time of dissection 12 weeks after the cell inoculation. The thoracic cavity was exposed and the heart root was clamped to arrest blood flow, after which an intraperitoneal injection (5 mL) of a mixture of 2.5% glutaraldehyde, 2% paraformaldehyde, and 0.01% sodium azide in 0.05 mol/L sodium cacodylate (pH 7.2) was given. After 10 min of primary fixation, the abdominal cavity was exposed and specimens, including peritoneal lining and jejunum (including mesenteries), were harvested. Specimens were further fixed overnight in the aldehyde mixture. After rinsing in 0.15 mol/L cacodylate, specimens for scanning electron microscopy (SEM) were subjected to an OTOTO postfixation procedure [39]. Dehydration followed in a series of ethanol, finally replaced by two changes of hexamethyldisalazane, which was allowed to evaporate under a fume hood. The dried specimens were mounted on aluminum stubs and were examined in a Zeiss 982 Gemini field emission scanning electron microscope after coating with palladium in an Emitech 550 sputter coater. Digital images were collected at a resolution of 1024 × 1024 pixels.

### Statistical Analysis

Logistic-regression models were set up in the R software (www.r-project.org) and used to evaluate the effects of treatment (kBq) and specific activity (^211^At atom/mAbs) on the probability of an animal remaining free from macroscopic tumors at the time of dissection. A model including specific activity, treatment and an interaction between treatment and specific activity did not, however, result in a significant improvement in explaining the probability of macroscopic tumors, than when only specific activity was included as explanatory variable (*P* > 0.77). Hence, the final model for the logistic regression included only the specific activity as explanatory variable.

## Results

**Figure 2:**
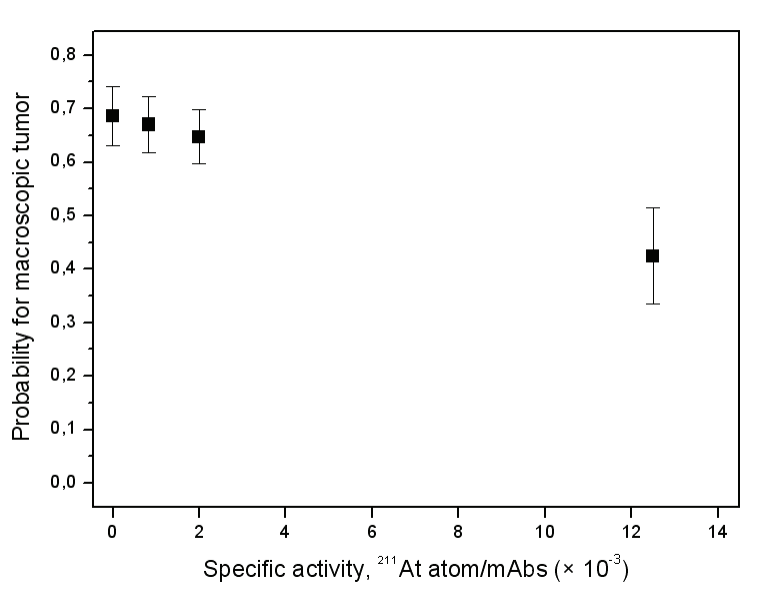
Probability for macroscopic tumor as a function of the specific activity (± SEM). Controls were given PBS (*n* = 10) or unlabeled MX35 F(ab′)2 in PBS (*n* = 10). Each specific activity level (*n* = 30) includes all 3 treatment levels (25, 50, and 400 kBq MX35 F(ab′)2).

The radiochemical yield following labeling of the MX35 F(ab′)2 was 80% resulting in a product with an activity concentration of 213 MBq/mL and a specific activity of 1920 MBq/mg corresponding to an antibody to ^211^At ratio of 84 : 1. The immunoreactivive fraction was excellent (95%) indicating retained biological function despite the very high specific activity. The therapeutic efficacy was estimated by determining the presence of macroscopic tumors by meticulous ocular examination of the abdominal cavity at the time of dissection. Summing over the different activity levels (25, 50, and 400 kBq ^211^At-MX35 F(ab′)2) the number of animals with macroscopic tumors were 13 (*n* = 30), 17 (*n* = 30), and 22 (*n* = 30) when treated with ^211^At-MX35 F(ab′)2 using specific activities equal to 1/80, 1/500, and 1/1200 ^211^At atoms/mAbs, respectively. Treatment with PBS (*n* = 10) or unlabeled MX35 F(ab′)2 in PBS (*n* = 10) resulted in only 3 out of 10 animals in each group being free from macroscopic tumors (Table 1). The logistic regression analysis showed a significant trend that higher specific activity means less probability for macroscopic tumors (*P* = 0.02) (Fig. 2). No significant difference in the probability for macroscopic tumors could be detected between the groups with different amount of activity (*P* > 0.05). Because the batch of animals we received for this study seemed to be more immunosuppressed than usual, the disease became very advanced and the presence of ascites and microscopic tumors were higher (100%) than expected at the time of dissection, irrespective of treatment regimen, and could therefore not lay any ground for estimating any differences in the therapeutic efficacy.

**Table 1:** Study Groups and Number of Mice with Macroscopic Tumors

Group	*n*	Treatment	Specific activity (At atom/mAbs)	Macroscopic tumors
1	10	25 kBq ^211^At-MX35 F(ab′)2 in PBS	l/80	5/10
2	10	50 kBq ^211^At-MX35 F(ab′)2 in PBS	l/80	4/10
3	10	400 kBq ^211^At-MX35 F(ab′)2 in PBS	l/80	4/10
4	10	25 kBq ^211^At-MX35 F(ab′)2 in PBS	l/500	7/10
5	10	50 kBq ^211^At-MX35 F(ab′)2 in PBS	l/500	5/10
6	10	400 kBq ^211^At-MX35 F(ab′)2 in PBS	l/500	5/10
7	10	25 kBq ^211^At-MX35 F(ab′)2 in PBS	l/1200	5/10
8	10	50 kBq ^211^At-MX35 F(ab′)2 in PBS	l/1200	9/10
9	10	400 kBq ^211^At-MX35 F(ab′)2 in PBS	l/1200	8/10
10	10	PBS	–	7/10
11	10	MX35 F(ab′)2 in PBS	–	7/10

**Figure 3:**
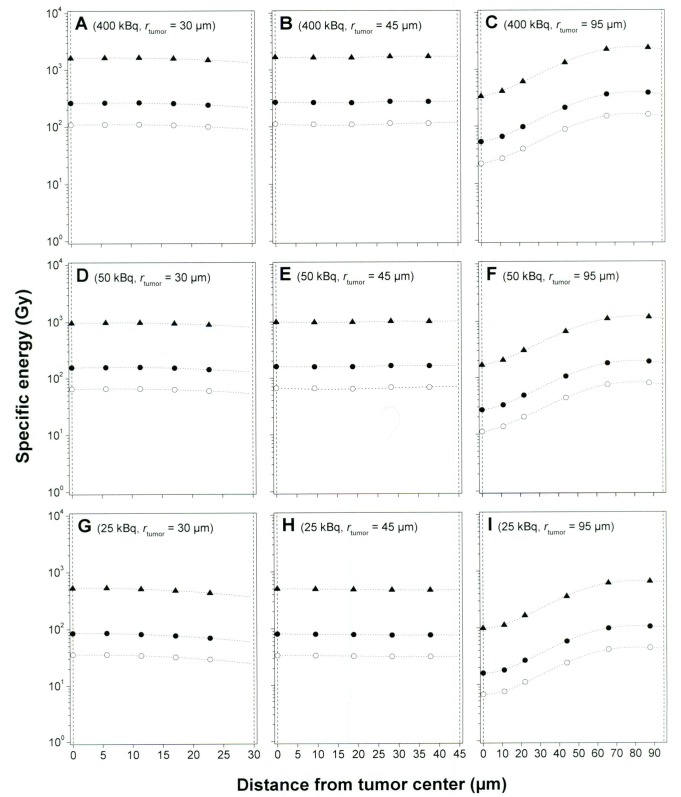
Specific energy imparted to a cell nucleus situated at different distances from the tumor center. The calculations were performed for 3 tumor sizes: 30, 45, and 95 µm radius; 3 activity levels: 25, 50, and 400 kBq ^211^At MX35 F(ab′)2; and 3 specific activity levels: 1/80 (▲), 1/500 (•), and 1/1200 (○) ^211^At atom/mAbs. A diffusion depth of 30 µm into each tumor was assumed for the radioactivity in all calculations. The vertical dashed lines in each panel represent the tumor center and surface, respectively.

The specific energy values shown in Figure 3 ranged from 7 Gy (25 kBq ^211^At MX35 F(ab′)2, *r*_tumor_ = 95 µm, and 1/1200 ^211^At atom/mAbs) to 1185 Gy (400 kBq ^211^At MX35 F(ab′)2, *r*_tumor_ = 95 µm, and 1/80 ^211^At atom/mAbs). For the assumed largest tumor (*r*_tumor_ = 95 µm) the specific energy to a cell nucleus situated in the core of the tumor increased from 22 to 334 Gy for 400 kBq ^211^At MX35 F(ab′)2 when using a specific activity of 1/80 instead of 1/1200, significantly increasing the probability for a cell kill. The successive decrease of the specific energy to cell nucleus situated closer to the core of the tumor is explained by the limited path length of the emitted α-particles.

**Figure 4:**
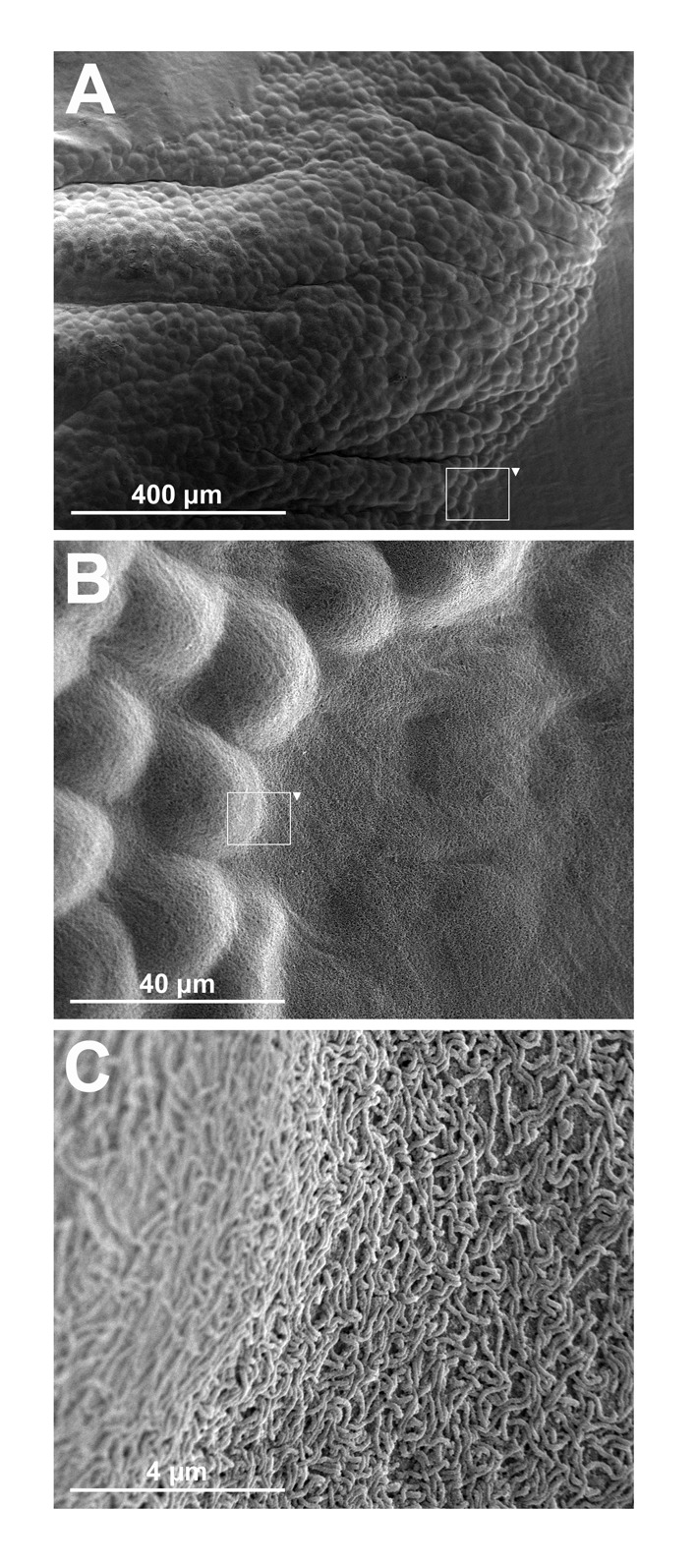
Scanning electron-microscopy images of macroscopic tumors on the peritoneum in nude mice at the time of dissection. Panel A shows a part of a macroscopic tumor several millimeters in length visible to the naked eye. Panel B shows an enlargement of a segment shown in panel A illustrating how the tumor cells disseminate under the mesothelial cell layer of the peritoneum. Panel C shows an enlargement of a segment shown in panel B, illustrating the microvilli covered transition from tumor to healthy tissue.

The findings on the peritoneal biopsies at the time of dissection revealed both larger tumor cell clusters of several millimetres in diameter as well as clusters consisting of only a few tumor cells. The tumors were sometimes only loosely adhered to the peritoneum, but were sometimes firmly anchored penetrating under the mesothelial cell layer (Fig. 4).

## Discussion

We have previously investigated the therapeutic efficacy of the intact specific IgG1 mAbs MOv18 and MX35 as well as fragmented mAbs (MX35 F(ab′)2 and non-specific Rituximab F(ab′)2) in the treatment of nude mice in an ovarian cancer model [22-27]. Those studies showed good therapeutic efficacy when injecting 400 kBq ^211^At-MX35 3 wk after cell inoculation, as well as the increasing importance of using a specific mAb compared to a non-specific mAb when treating larger tumors. In the interval of 400-1200 kBq the therapeutic efficacy was not correlated with the administered activity. This could be explained by the saturation of the antigenic sites, which-according to the dynamic compartmental model used in both those and this study-occurs within a few hours after the injection, resulting in a similar absorbed dose for the 3 activity levels of 400, 800, and 1200 kBq. The rationale for choosing the fragmented mAb instead of the whole IgG is due to 4 facts: (*i*) The fragmented mAb was the only clinical grade version of the mAb available at the time of the study; (*ii*) We have received an approval by the Swedish Medical Products Agency for completing a phase I study with this fragmented mAb [33]; (*iii*) We believe that the diffusion into tumors using the fragmented mAb is higher than compared to whole IgG; and (*iv*) We believe that the immunogenicity of the fragmented mAb is lower than the whole IgG, reducing the risk for HAMA response, especially if repeated treatments are considered in the future.

As shown in one of our earlier studies there will be tumors with maximum radii of 95 µm when injecting the radioimmunocomplex 4 wk after tumor cell inoculation [27]. This means that the core of the largest tumors will not be irradiated if the activity is only situated on the surface of the tumors. However, if we have a diffusion of the radioimmunocomplex of 30 µm in the tumors the core will be irradiated. A highest activity of 400 kBq was chosen in this study based on two facts; (*i*) it is well within the toxic limit [2]; (*ii*) further increase in the activity does not improve the therapeutic efficacy. In those earlier studies the specific activity had been 1/1200 ^211^At atom/mAbs.

As we just recently have published a phase I study on women with recurrent ovarian cancer, and now prepare to initiate a phase II study, we find it of outmost importance to investigate the role of higher specific activities [33]. The results from the phase I study, indicated no adverse effects of the treatment, using intraperitoneally injected ^211^At labeled MX35 F(ab′)2. This is very encouraging and urges us to initiate the phase II study now under planning. The choice of the 3 levels of specific activity in this study (i.e. 1/80, 1/500, and 1/1200) was based on the fact that: (i) 1/80 was for the moment the highest achievable specific activity; (ii) 1/1200 has up until recently been the highest specific activity achievable; and (iii) 1/500 was chosen as an intermediate value between 1/80 and 1/1200.

The advantages with administering a radioimmunocomplex with a high specific activity could be that: (i) able to treat low antigen expressing tumor and/or tumors having restricted diffusion; and (ii) able to treat more patients per unit radioactivity. The number of antigens on NIH:OVCAR-3 cells, *B*_max_, determined *in vitro* are 10^6^. What *B*_max_ will be for the patients in for example the forthcoming phase II study is not known, but it will most probably vary. Therefore, it is important to administer a radioimmunocomplex with as high specific activity as possible. Since the availability of ^211^At still is limited it is important to use the accessible amount of radioactivity as effectively as possible, implying the use of a high specific activity lowering the necessary total amount of administered radioactivity.

If there are insurmountable problems using only α-particle emitters when trying to treat a disseminated disease where both micro- and macrotumors are present, and for which there exists an unmanageable diffusion problem for the effector molecules into the tumors, and for which a pre-targeting system does not work satisfactory, the use of a cocktail of both α-particle and electron-emitters could be the solution. In our situation with ovarian cancer the combination of an intraperitoneal as well as an intravenous administration route could also be relevant, if there are larger vascularized extraperitoneally tumors present at the time of treatment.

As already mentioned in the Results the batch of animals we received for this study seemed to be more immunosuppressed than usual. The disease therefore became very advanced and the presence of ascites and microscopic tumors were much higher (100%) than compared to earlier studies at the time of dissection, and could therefore not lay any ground for estimating any differences in the therapeutic efficacy. However, we find the results of the study of outmost importance, indicating the role of the specific activity even when the disease is advanced, and believe it therefore very important to publish these results. A series of studies are now planned to further investigate the role of the specific activity for various levels of the specific activity, amounts of injected activity, and degree of advanced disease.

In conclusion, summing over the different activity levels (25, 50, and 400 kBq ^211^At-MX35 F(ab′)2) the number of animals with macroscopic tumors was 13, 17, and 22 (*n* = 30 for each group) for the specific activities equal to 1/80, 1/500, or 1/1200, respectively. Logistic-regression analysis showed a significant trend that higher specific activity imply a decreased probability for macroscopic tumors at the time of dissection (*P* = 0.02). Increasing the specific activity indicates a way to enhance the therapeutic outcome. Further studies of the role of the specific activity are therefore important and justified.
